# The timing and relationship of ventricular arrhythmia with exercise patterns in veteran male endurance athletes

**DOI:** 10.1093/eurjpc/zwag021

**Published:** 2026-01-12

**Authors:** Wasim Javed, Benjamin Brown, Bradley Chambers, Eylem Levelt, Lee Graham, John P Greenwood, Sven Plein, Peter P Swoboda

**Affiliations:** Multidisciplinary Cardiovascular Research Centre and Department of Biomedical Imaging Science, Leeds Institute of Cardiovascular and Metabolic Medicine, University of Leeds/Leeds Teaching Hospitals National Health Service Trust, Clarendon Way, Leeds LS2 9JT, UK; Multidisciplinary Cardiovascular Research Centre and Department of Biomedical Imaging Science, Leeds Institute of Cardiovascular and Metabolic Medicine, University of Leeds/Leeds Teaching Hospitals National Health Service Trust, Clarendon Way, Leeds LS2 9JT, UK; Multidisciplinary Cardiovascular Research Centre and Department of Biomedical Imaging Science, Leeds Institute of Cardiovascular and Metabolic Medicine, University of Leeds/Leeds Teaching Hospitals National Health Service Trust, Clarendon Way, Leeds LS2 9JT, UK; Department of Cardiometabolic Imaging, Baker Heart and Diabetes Institute & University of Melbourne, Melbourne, Australia; Multidisciplinary Cardiovascular Research Centre and Department of Biomedical Imaging Science, Leeds Institute of Cardiovascular and Metabolic Medicine, University of Leeds/Leeds Teaching Hospitals National Health Service Trust, Clarendon Way, Leeds LS2 9JT, UK; Department of Cardiometabolic Imaging, Baker Heart and Diabetes Institute & University of Melbourne, Melbourne, Australia; Multidisciplinary Cardiovascular Research Centre and Department of Biomedical Imaging Science, Leeds Institute of Cardiovascular and Metabolic Medicine, University of Leeds/Leeds Teaching Hospitals National Health Service Trust, Clarendon Way, Leeds LS2 9JT, UK; Multidisciplinary Cardiovascular Research Centre and Department of Biomedical Imaging Science, Leeds Institute of Cardiovascular and Metabolic Medicine, University of Leeds/Leeds Teaching Hospitals National Health Service Trust, Clarendon Way, Leeds LS2 9JT, UK

**Keywords:** Ventricular arrhythmia, Sports cardiology, Exercise tracking, Veteran athletes

## Abstract

**Aims:**

The aim of this study was to determine whether exercise training patterns were associated with the incidence and timing of ventricular arrhythmia in veteran male endurance athletes.

**Methods and results:**

One-hundred and six healthy male endurance athletes (cyclists/triathletes) aged ≥50 y undertaking ≥10 h/week of exercise for ≥15 y underwent clinical assessment, cardiac magnetic resonance (CMR), and implantable loop recorder (ILR) implantation. Daily exercise was tracked with computerized exercise tracking devices. Athletes were followed up for ventricular arrhythmia on ILR, ventricular tachycardia (VT), and non-sustained VT (NSVT). Fifty-five ventricular arrhythmia events occurred (median follow-up 796 days): 3 (5.5%) VT and 52 (94.5%) NSVT in 25 (23.5%) athletes. Myocardial fibrosis was significantly more prevalent in athletes with ventricular arrhythmia than those without ventricular arrhythmia [19 (76.0%) vs. 31 (38.3%), *P* < 001]. The incidence of exercise-related ventricular arrhythmia was 0.4/1000 h of exercise vs. non-exercise-related ventricular arrhythmia incidence of 0.01/1000 h of non-exercise. All three sustained VT cases occurred during exercise in athletes with fibrosis and were preceded by NSVT. There were no training differences between athletes with and without ventricular arrhythmia over 2 years and in the month prior to each arrhythmic event.

**Conclusion:**

A significant proportion of highly trained male veteran athletes developed ventricular arrhythmia which was predominantly NSVT and was strongly associated with myocardial fibrosis. Acute exercise exposure was associated with an increased risk of developing ventricular arrhythmia, but chronic exercise load was not. Our findings therefore highlight myocardial fibrosis as a potential pro-arrhythmic substrate upon which intense exercise may trigger arrhythmogenesis in certain male veteran athletes.

## Graphical Abstract

**Graphical Abstract:**
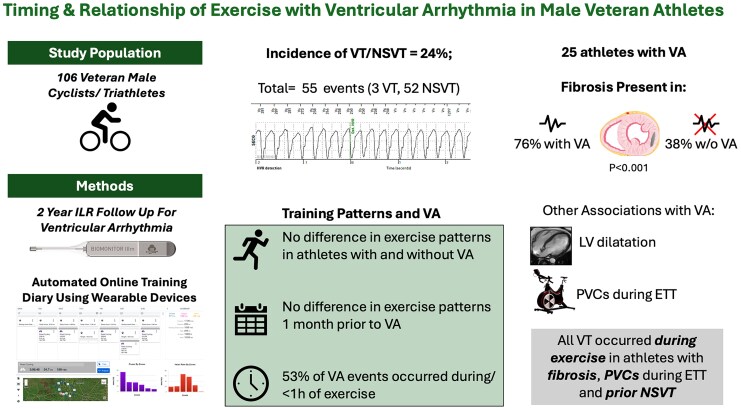

## Introduction

The cardiovascular benefits of moderate habitual exercise are undisputed.^1^ However, sudden cardiac death (SCD) is a leading cause of mortality in athletes which predominantly affects older male recreational athletes.^2^ In a significant proportion of athletes who die from SCD, a structurally normal heart is found during post-mortem examination and primary arrhythmia is deemed likely responsible in these cases.^3^ The circumstances surrounding fatal ventricular arrhythmogenesis in athletes are uncertain. Traditionally, a ‘perfect storm’ scenario has been postulated where a combination of pro-adrenergic factors related to competitive exercise including tachycardia, catecholamine release, stimulant usage along with dehydration, and electrolyte imbalance may trigger ventricular arrhythmogenesis in the presence of an arrhythmic substrate such as cardiomyopathy or channelopathy.^4^ However, it is unclear whether this is also applicable to those with athletically adapted hearts, where no obvious substrate is present, as opposed to those with underlying cardiac disease.

Most studies investigating the adverse cardiovascular effects of exercise have relied upon self-reported training histories which may be inaccurate. Furthermore, it is not known whether exercise activity, measured comprehensively, accurately, and prospectively, influences the incidence of ventricular arrhythmia in older athletes in whom exercise-induced cardiac adaptations are likely to be more long-standing. Among many current endurance athletes, there is widespread usage of cycling computers and/or global positioning system (GPS) smartwatches to comprehensively monitor power output, heart rate (HR), training volume, intensity, and frequency along with rest periods. Whilst athletes primarily utilize these devices to track their exercise training with the aim of improving performance, they also provide an accurate training log for research purposes. By combining the use of these comprehensive exercise tracking devices (CETDs) with concurrent monitoring for ventricular arrhythmia, it is possible to examine the direct relationship of exercise with ventricular arrhythmia along with the circumstances and timing of ventricular arrhythmia.

We aimed to prospectively establish whether the exercise training habits of veteran endurance athletes measured using CETDs were associated with the incidence and timing of ventricular arrhythmia on long-term implantable loop recorder (ILR) monitoring. Furthermore, we aimed to characterize the sporting and clinical details associated with the onset of individual arrhythmic events.

## Methods

### Study recruitment and design

This research was undertaken in accordance with the Declaration of Helsinki. It was granted ethical approval by the South Yorkshire & Humber NHS Research Ethics Committee and Health Research Authority (21/YH/0231), IRAS project ID: 300629. Local ethical approval was also obtained from Leeds Teaching Hospitals NHS Trust for the study. Each participant provided written informed consent prior to taking part in the research.

Athletes in this study were part of VENTOUX; a prospective study which recruited 106 competitive veteran endurance athletes (cyclists/triathletes) via email invitation to their respective club/organization within the UK.^5^ Inclusion criteria consisted of being a male cyclist or triathlete aged over 50 years old and exercised for 10 or more hours per week for at least 15 years.

Exclusion criteria consisted of pre-existing cardiovascular disease (CVD) (including coronary artery disease, tachyarrhythmia, and arterial hypertension) or any significant medical condition (mild hyperlipidaemia defined as requiring the use of a single lipid lowering medication and without a known inherited lipid disorder and mild asthma were not considered significant). Athletes were excluded if they had any cardiac symptoms suggestive of underlying CVD (anginal chest pain, palpitations, exertional dyspnoea, and syncope).

Participants underwent clinical assessment, 12-lead ECG, cardiac magnetic resonance (CMR), exercise testing, and ILR implantation. Participants utilized a CETD during exercise which automatically transferred exercise data to an online training diary.

### Baseline assessment

All participants underwent baseline assessment consisting of physical examination which included measurement of resting blood pressure (BP) and HR. A self-reported full medical, sporting, and lifestyle history was documented, and cardiovascular risk factors were recorded including any prior history of hyperlipidaemia and family history of CVD and SCD. Social histories were taken consisting of smoking status and alcohol and caffeine intake along with any use of performance enhancing drugs. All participants underwent resting 12-lead ECG (MAC500, GE Medical Systems, Milwaukee, WI, USA).

### Cardiac magnetic resonance

All participants underwent comprehensive stress-perfusion CMR with late gadolinium enhancement (LGE) of which the protocol has been previously described.^5^ Briefly, this consisted of cine imaging, adenosine stress perfusion, T1 and T2 mapping, and LGE imaging.

### Exercise testing

Participants underwent a supervised exercise cycling test on a stationary exercise bicycle (Wattbike Pro, Wattbike, UK) after abstaining from caffeine and strenuous exercise for 24 h prior. Exercise tests consisted of a 5-min freestyle cycling warm-up followed by a maximal ramp-incremental test and ended with a 5-min low intensity recovery ride. Starting power was determined by weight and competition level according to manufacturer’s product guidance with incremental increases of 20 W per minute until complete exhaustion was reached.

Throughout the test, cadence, power output, and HR were recorded to enable calculation of functional threshold power (FTP) output. Functional threshold power provides the estimated maximum power attainable for 1 h and may correlate better with cycling performance than traditional VO_2_max testing in certain endurance athletes.^6^ The maximal power output in the final minute prior to exhaustion (MMP) was recorded and from this FTP values were calculated by MMP multiplied by 0.75. Maximum HR, estimated maximal oxygen consumption (VO_2_max), and metabolic equivalent of tasks (METs) were also recorded. Continuous ECG monitoring was utilized throughout to detect the presence of premature ventricular contractions (PVCs). Athletes were classified as having PVC during exercise if they exhibited ≥1 PVC during the exercise test. Premature ventricular contractions were characterized as atypical if they were multifocal, demonstrated R-on-T phenomena, or occurred in couplets or greater.

### Implantable loop recorder

Each participant received a Biomonitor IIIm (Biotronik GmBH & Co, Berlin Germany) ILR. Implantable loop recorders were programmed to record tachyarrhythmia faster than the participant’s maximum HR defined on exercise testing for ≥8 consecutive beats. Implantable loop recorders contained accelerometers to measure general physical activity. Automated nightly remote downloads were performed by a CardioMessenger Smart mobile unit (Biotronik GmBH & Co, Berlin Germany) that each participant was provided with. Implantable loop recorders were left in place for the entirety of the study unless there was a clinical indication or participant request for removal.

Each participant was asked to continue with their normal daily activities and sporting habits. Participants recorded any symptoms via the Biotronik Patient Application (Biotronik GmBH & Co, Berlin Germany) which was downloaded on participant’s smartphones at the time of ILR implantation.

### Follow-up

Participants were followed up with daily monitoring of ILR heart rhythm data for the main outcome of ventricular arrhythmia. Ventricular tachycardia (VT) was defined as equal to or greater than 30 s of consecutive ventricular beats at equal to or greater than 100 b.p.m., and non-sustained ventricular tachycardia (NSVT) was defined as equal to or greater than three consecutive ventricular beats lasting less than 30 s as per the European Society of Cardiology (ESC) 2022 ‘The management of patients with ventricular arrhythmias and the prevention of SCD’ guidelines.^2^ All arrhythmic events were confirmed by a consultant electrophysiologist. Any participant who developed a potentially harmful arrhythmia was contacted urgently to assess symptoms and advise them to seek independent medical attention if appropriate. Participants were sent 6-monthly questionnaires to complete which detailed any new medical diagnoses or medication commenced.

### Exercise activity

Participants received instructions on accessing an online exercise diary (Training Peaks, Louisville, USA) at the time of ILR implantation. They synchronized their CETD with this online training to enable automated exercise tracking and were advised to continue their normal exercise habits. Comprehensive exercise tracking devices included cycling computers and GPS smart watches in addition to cycling power metres (when cycling) and chest-worn HR monitors.

Throughout the study, we accessed the online training diary of each athlete. Recorded data included the type of exercise performed along with exercise frequency, duration, and distance travelled. Exercise intensity was also recorded with a proprietary formula-based method known as training stress score (TSS). Training stress score was automatically calculated based on an individual’s relative exercise load and duration of each exercise session. In this formula, exercise intensity was calculated by dividing an individual’s normalized power during an exercise session by their FTP obtained during exercise testing which is considered a non-invasive alternative to lactate threshold (LT).^7^

When exercise activity involved cycling, athletes utilized a cycling computer in combination with a power metre to produce power output data from which six power zones were categorized. These power zones were based on Allen and Coggan’s power zones which represent specific physiological training functions with increasing workloads based on an individual’s FTP (*Table 1*).^8^

**Table 1:** Power/heart rate zone classification

Zone	Percentage of FTP/MHR (%)	Specific training function
1	<55	Active recovery
2	55–74	Endurance
3	75–89	Tempo
4	90–104	Threshold
5	105–120	V0_2_ max
6	>120	Anaerobic capacity and neuromuscular power

FTP, functional threshold power; MHR, maximum heart rate; V0_2_ max, maximum oxygen consumption.

Heart rate during exercise was also recorded via the participant’s own HR monitor equipment (GPS watch or chest-worn device). Heart rate zone data were then automatically calculated in the same method of power zones but in relation to maximum HR during exercise which was determined by the exercise test at the time of study visit.

Exercise data were collected over the entire follow-up period allowing for total exercise to be calculated along with one month prior to the onset of any ventricular arrhythmic event and the specific exercise session immediately prior to an arrhythmic event.

### Statistical analysis

Statistical analyses were undertaken using SPSS statistics 29 (IBM SPSS, Armonk, New York, USA). Any athlete with a training diary gap of over 1 month was excluded from the analysis. Normality of data was assessed using Shapiro–Wilk test. Continuous data were presented as mean ± standard deviation or median ± interquartile range (IQR) depending on the normality of the data. Categorical data were presented as frequency (%). Continuous variables were compared using unpaired *t*-test or Mann–Whitney U-test depending on the normality of data. Categorical variables were compared using *χ*² test or Fisher exact test depending on the number of observed events. Depending upon normality of data, either Pearson’s or Spearman’s correlation coefficient was used to assess correlation. Analysis of variance (ANOVA) with *post hoc* Bonferroni correction was used to assess differences between multiple groups. A *P* value of less than 0.05 was considered statistically significant in all analyses.

## Results

Ninety-seven (91.5%) athletes completed the study [median follow-up 796 days (IQR 741–832 days). Nine (8.4%) athletes either requested to have their ILR devices removed during follow-up (six athletes, median 123 days) or did not adequately comply with exercise tracking over the course of the study (three athletes).

### Incidence of ventricular arrhythmia

Twenty-five (23.5%) athletes experienced at least one ventricular arrhythmic episode over the follow-up period of which three (2.8%) athletes developed VT and 22 (20.8%) athletes experienced NSVT. A total of 55 ventricular arrhythmic episodes occurred with three (5.5%) episodes of VT and 52 (94.5%) episodes of NSVT. All episodes of VT were preceded by NSVT and were symptomatic in contrast to NSVT episodes which were all asymptomatic. The median length of ventricular arrhythmia was 10 beats (IQR 3.0–25.0 beats).

There were no significant differences in baseline characteristics in athletes who developed ventricular arrhythmia and those who did not. However, athletes who experienced ventricular arrhythmia reported a significantly lower number of previous training years [20.0 years (13.3–30.0) vs. 15.0 years (10.0–20.0), *P* = 0.04] with no differences in training hours per week or competitions per year (*Table 2*). During exercise testing, athletes with ventricular arrhythmia had a greater prevalence of PVCs [21 (84.0%) vs. 35 (47.3%) *P* = 0.001] with atypical features [14 (56.0%) vs. 11 (14.9%), *P* = 0.003] but no difference in FTP (250.2 ± 29.0 vs. 239.9 ± 29.0, *P* = 0.14) than athletes without ventricular arrhythmia. On CMR, athletes with ventricular arrhythmia had a significantly greater prevalence of myocardial fibrosis [19 (76.0%) vs. 31 (38.3%), *P* < 0.001] and greater LVEDVi (mL/m^2^) (114 ± 17 vs. 106 ± 13, *P* = 0.02) than athletes who did not experience ventricular arrhythmia. Comparison of athletes with ventricular arrhythmia according to the presence of myocardial fibrosis can be found within the Supplementary material (Supplementary material online, *Table S1*).

**Table 2:** Baseline demographic and training characteristics comparing athletes with and without ventricular arrhythmia

	No VA (*n* = 81)	VA (*n* = 25)	*P* value
Baseline characteristics			
Age (years)	59.0 ± 5.6	60.0 ± 5.7	0.43
Body mass index (kg/m^2^)	24.8 ± 2.8	25.2 ± 2.6	0.56
Systolic blood pressure (mmHg)	121.1 ± 10.7	117.2 ± 10.5	0.10
Diastolic blood pressure (mmHg)	75.0 ± 7.1	72.7 ± 7.3	0.17
Resting heart rate (b.p.m.)	54.1 ± 7.5	51.9 ± 5.5	0.18
Smoker (*n*)	2 (2.5%)	0	0.43
Ex-smoker (*n*)	17 (21.0%)	2 (8.0%)	0.14
Alcohol intake (units/week)	7.5 (2.0–17.5)	5.0 (0.5–10.0)	0.21
Hyperlipidaemia (*n*)	2 (2.5%)	2 (8.0%)	0.21
Self-reported training history			
Training years (>10 h/week)	20.0 (13.3–30.0)	15.0 (10.0–20.0)	0.04*
Weekly training (hours)	11.9 ± 3.4	11.0 ± 2.1	0.40
Competitions per year	20.0 (10.0–30.0)	20.0 (12.8–26.3)	1.00
12-lead ECG			
Premature ventricular contraction (*n*)	0	3 (12.0%)	0.002*
Anterior T-wave inversion (*n*)	1 (1.2%)	1 (4.0%)	0.42
Lateral T-wave inversion (*n*)	1 (1.2%)	2 (8.0%)	0.08
Anterior Q-wave (*n*)	2 (2.5%)	0	0.43
Lateral Q-wave (*n*)	5 (6.2%)	3 (12.0%)	0.34
LBBB	1 (1.2%)	0	0.58
RBBB	1 (1.2%)	1 (4.0%)	0.37
Low voltage QRS	17 (21.0%)	3 (12.0%)	0.32
LVH voltage criteria	19 (23.5%)	6 (24.0%)	0.96
Exercise test			
FTP (W)	239.9 ± 29.0	250.2 ± 29.0	0.14
Maximum heart rate (b.p.m.)	168.5 ± 11.7	162.7 ± 13.6	0.05
PVCs during exercise testing (*n*)	35 (47.3%)	21 (84.0%)	0.001*
Number of PVCs during exercise testing when present (beats)	4 (1–7)	10 (1–23)	0.20
Atypical PVCs during exercise testing (*n*)	11 (14.9%)	14 (56.0%)	0.003*
Prospective exercise data			
ILR physical activity (%/day)	20.1 ± 4.3	20.6 ± 3.7	0.40
Pure cyclist (*n*)	60 (82.2%)	19 (79.2%)	0.74
Weekly total exercise (hours)	7.2 ± 3.1	7.2 ± 3.0	0.93
Weekly cycling distance (miles)	117.2 ± 57.3	110.0 ± 46.1	0.58
Weekly exercise intensity (TSS)	338 (229–484)	366 (284–448)	0.98
Monthly exercise frequency (*n*)	14.5 (10.2–18.8)	16.4 (12.6–20.1)	0.63
Annual cycling distance (miles)	6094 ± 2979	5722 ± 2400	0.58
CMR			
LVEDVi (mL/m^2^)	106 ± 13	114 ± 17	0.02*
LVEF (%)	56.0 ± 4.2	55.7 ± 4.2	0.80
LVMi (g/m^2^)	69.7 ± 9.5	72.0 ± 10.0	0.30
RVEDVi (mL/m^2^)	109 ± 17	114 ± 17	0.20
RVEF (%)	53.2 ± 5.2	52.1 ± 6.4	0.38
Fibrosis (*n*)	31 (38.3%)	19 (76.0%)	<0.001*
RVIP LGE (*n*)	58 (71.6%)	21 (84.0%)	0.21
Native T1 (ms)	1241 ± 38	1250 ± 47	0.33
ECV (%)	21.1 ± 2.1	21.1 ± 2.0	0.98
T2 (ms)	40.3 ± 1.8	40.9 ± 2.2	0.17

b.p.m., beats per minute; FTP, functional threshold power; ILR, implantable loop recorder; PVC, premature ventricular contraction; TSS, training stress score; VA, ventricular arrhythmia (PVCs during exercise testing; no VA = 74, VA = 25).

Bold values * denote *P* < 0.05.

**Figure 1:**
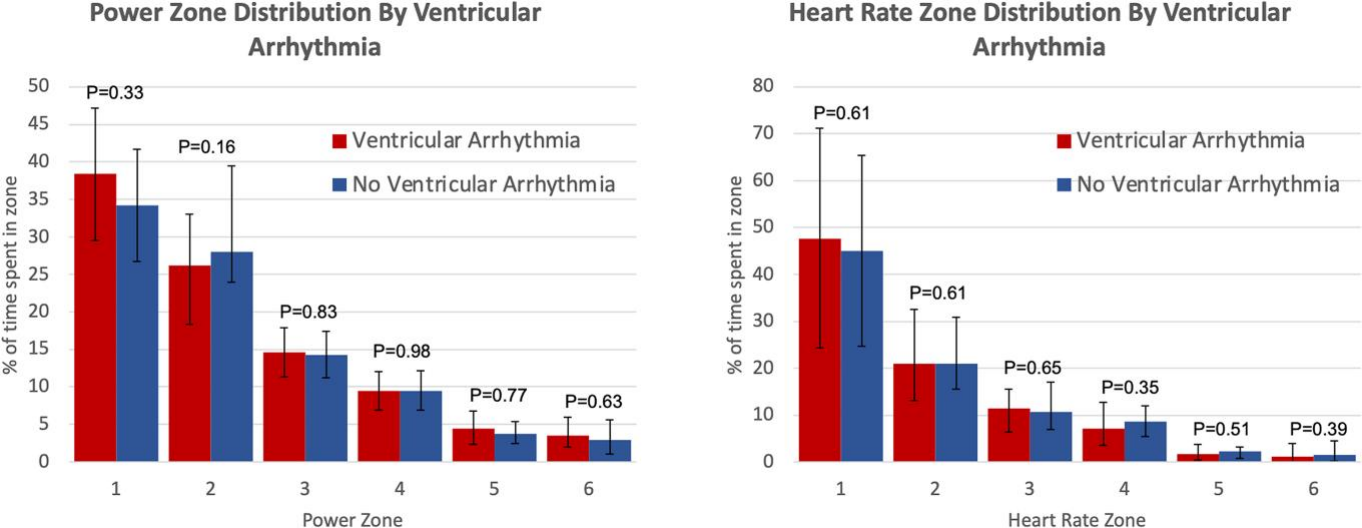
Comparison of exercise session power and heart rate zones between athletes with and without ventricular arrhythmia. Red bars indicate athletes with ventricular arrhythmia, and blue bars indicate athletes without ventricular arrhythmia.

There were no significant differences in prospective exercise duration (7.2 ± 3.0 h/week vs. 7.2 ± 3.1 h/week, *P* = 0.93), distance (110.0 ± 46.1 miles/week vs. 117.2 ± 57.3 miles/week, *P* = 0.58), or intensity (TSS) [366.2 (283.5–447.5) vs. 338.0 (228.7–483.8), *P* = 0.98] between athletes with and without ventricular arrhythmia. Furthermore, there were no differences in relation to whether an athlete was a cyclist or triathlete and the time spent in specific power and HR zones between athletes who did and did not experience ventricular arrhythmia (*Figure 1*).

Of those three athletes who developed sustained VT, they all had myocardial fibrosis on CMR and all exhibited PVCs with atypical features during exercise testing. These athletes underwent clinical evaluation by their local consultant cardiologist. Of those, one received an implantable cardiac defibrillator (ICD) due to pre-syncope, one was scheduled for an electrophysiology (EP) study, and the third was advised to cease competing due to recurrent VT during exercise but declined further investigation.

### Exercise patterns in the month prior to ventricular arrhythmia

When comparing exercise variables over 2 years with the month prior to the onset of a ventricular arrhythmic event, there were no significant differences in exercise duration [30.3 (23.9–37.0) hours ± 31.5 (22.4–43.9) hours, *P* = 0.13], distance [480.8 (354.8–537.0) miles vs. 483.3 (263.2–632.5) miles, *P* = 0.22], or intensity (TSS) [1525.9 (1340.9–2139.8) vs. 1849.0 (1443.8–2462.8), *P* = 0.36] (*Table 3*). There were also no significant differences in the time spent in the highest power and HR zones between the time intervals.

**Table 3:** Training patterns in the month before ventricular arrhythmia

	Monthly exercise (*n* = 55 events)	1 month prior to VA (*n* = 55 events)	*P* value
Exercise frequency (*n*)	16.8 (13.3–20.2)	21.0 (14.3–27.8)	0.12
Exercise duration (hours)	30.3 (23.9–37.0)	31.5 (22.4–43.9)	0.13
Exercise distance (miles)	480.8 (354.8–537.0)	483.3 (263.2–632.5)	0.22
Exercise intensity (TSS)	1525.9 (1340.9–2139.8)	1849.0 (1443.8–2462.8)	0.36
Power zones (% of time spent)			
1	29.8 (13.9–41.6)	32.8 (15.0–42.7)	0.51
2	28.0 (23.6–41.2)	28.4 (21.7–35.7)	0.58
3	14.1 (8.2–20.5)	15.1 (11.3–21.8)	0.04*
4	9.7 (5.2–12.3)	9.4 (7.0–14.0)	0.94
5	3.8 (2.4–6.1)	4.6 (2.8–7.1)	0.25
6	3.6 (1.0–6.1)	4.0 (1.6–8.2)	0.68
Heart rate zones (% of time spent)			
1	55.7 (42.3–62.0)	50.4 (38.0–69.8)	0.65
2	20.9 (13.2–24.7)	15.8 (11.3–21.4)	0.02*
3	10.2 (6.1–13.7)	9.2 (4.6–15.8)	0.43
4	6.6 (5.4–10.0)	7.5 (2.5–14.4)	0.95
5	1.6 (1.1–2.8)	1.2 (0.1–3.7)	0.95
6	2.6 (0.4–4.4)	1.3 (0.0–8.2)	0.07

TSS, training stress score; VA, ventricular arrhythmia (*n* = 41 for power zones, *n* = 54 for heart rate zones).

Bold values * denote *P* < 0.05.

### The timing of ventricular arrhythmia in relation to exercise

Of the 55 ventricular arrhythmic events, 29 (52.7%) events occurred during or within 1 h of exercise, 14 (25.5%) events occurred between one to 24 h since the last exercise session, and 12 (21.8%) events occurred 24 h after exercise was last performed. Furthermore, five (9.1%) ventricular arrhythmic episodes were nocturnal.

The incidence of exercise-related ventricular arrhythmia was 0.4/1000 h of exercise vs. non-exercise-related ventricular arrhythmia incidence of 0.01/1000 h of non-exercise (relative risk = 28.6 times). In terms of exercise variables related to the specific timing of ventricular arrhythmia, there were no significant differences in the exercise duration, distance, or intensity of the last training session (*Table 4*):

- Duration [<1 h since exercise; 0.9 (0.6–1.3) h vs. 1–24 h since exercise; 1.2 (0.5–1.7) h vs. >24 h since exercise 2.3 (0.5–2.8) h, *P* = 0.53]
- Distance [<1 h since exercise; 16.2 (6.9–23.4) miles vs. 1–24 h since exercise; 18.2 (11.5–36.5) miles vs. >24 h since 34.2 (6.8–43.0) miles, *P* = 0.53]
- Intensity (TSS) [<1 h since exercise; 93.2 ± 60.1 vs. 1–24 h since exercise; 94.2 ± 58.7 vs. >24 h since 120.1 ± 96.6, *P* = 0.57]

**Table 4:** Relationship of exercise with timing of ventricular arrhythmia

	Time since last exercise session to onset of VA			
	< 1 h (*n* = 29)	1–24 h (*n* = 14)	> 24 h (*n* = 12)	*P* value
Last exercise session duration (h)	0.9 (0.6–1.3)	1.2 (0.5–1.7)	2.3 (0.5–2.8)	0.53
Last exercise session distance (miles)	16.2 (6.9–23.4)	18.2 (11.5–36.5)	34.2 (6.8–43.0)	0.53
Last exercise session intensity (TSS)	93.2 ± 60.1	94.2 ± 58.7	120.1 ± 96.6	0.57
Exercise frequency (*n*)	25.2 ± 17.6	14.2 ± 5.3	19.0 ± 11.8	0.07
PVC during exercise testing (*n*)	26 (89.7%)	12 (85.7%)	8 (66.7%)	0.19
Significant bradyarrhythmia on ILR (*n*)	10 (34.5%)	9 (64.3%)	6 (50%)	0.18

ILR, implantable loop recorder; PVC premature ventricular complex; TSS, training stress score; VA, ventricular arrhythmia.

Furthermore, the prevalence of PVCs during exercise testing was greater in athletes who experienced ventricular arrhythmia during or within 1 h of exercise [26 (89.7%)] compared with athletes who experienced ventricular arrhythmia over 24 h since exercise [8 (66.7%)] although this did not reach statistical significance (*P* = 0.19) (*Table 4*).

**Figure 2:**
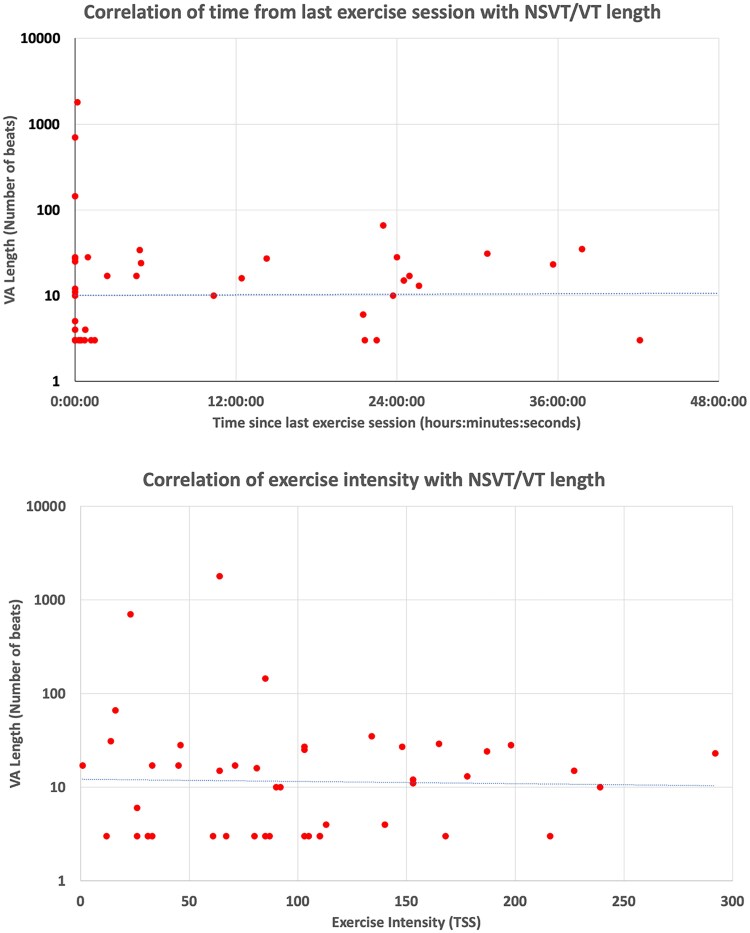
Scatter plots demonstrating relationship of length of ventricular arrhythmia with time since last exercise session (top) and training intensity (bottom). Time from last exercise session was filtered at ≤48 h. Red dots indicate events plotted on length of ventricular arrhythmia (*y* axis both graphs) by time since last exercise session (*x* axis top graph—*R* = 0.35, *P* = 0.009) and exercise intensity (*x* axis bottom graph—*R* = −0.01, *P* = 0.94). Line of best fit is indicated by grey horizontal line.

All three cases of VT occurred during or within 1 h of exercise activity. However, on Spearman’s rank correlation, there was no correlation between ventricular arrhythmia length and time since exercise (*R* = 0.35, 95% CI 0.09–0.57, *P* = 0.009) nor exercise intensity (*R* = −0.01, *P* = 0.94) (*Figure 2*).

## Discussion

In this study, we comprehensively tracked training habits using CETDs in healthy veteran male athletes whilst simultaneously monitoring the incidence of ventricular arrhythmia on ILR over 2.2 years. The vast majority of ventricular arrhythmic events were asymptomatic NSVT which has been associated with adverse outcomes in healthy middle and older-aged healthy individuals.^9^

The cumulative incidence of ventricular arrhythmia in our study was 23.5% over 2.2 years. Similar studies utilizing long-term continuous cardiac rhythm monitoring in unselected athletes are lacking. A study involving Italian athletes which followed up young athletes (82% male; mean age 28 years) with prior exercise-induced PVCs or NSVT found an incidence of sustained ventricular arrhythmia of 4% over 6.2 years.^10^ Comparatively, our incidence of sustained ventricular arrhythmia was 2.8% over 2.2 years. Other studies have not prospectively followed up athletes for the incidence of ventricular arrhythmia but have utilized 24 h ECG monitoring. Graziano *et al*. demonstrated a prevalence of 2% of NSVT in 433 healthy competitive athletes (mean age 27 years) whilst Zorzi *et al.* found a NSVT prevalence of 3% in 134 healthy competitive athletes (median age 45).^11,12^ However, both of these studies demonstrated that ventricular arrhythmia was significantly associated with increasing age.

The relatively high incidence of ventricular arrhythmia in our study may be due to the fact that it involved veteran male athletes with a high life-long exposure to high-intensity exercise and were continuing to exercise at a similar level throughout the study. Furthermore, the use of continuous ECG monitoring rather than short periods of Holter ECG monitoring is likely to have also contributed to this increased incidence.

### Training factors associated with ventricular arrhythmia

Overall, the type, amount, and intensity of exercise did not differ between athletes with and without ventricular arrhythmia suggesting that differing levels of exercise activity did not influence the incidence of ventricular arrhythmia in our cohort. These findings are in keeping with a study by Andersen *et al.* which investigated the incidence of arrhythmia in male cross-country skiers and found no association between finishing time and number of completed races with ventricular arrhythmia.^13^ However, our findings may have been influenced by including participants who undertook similarly high levels of exercise making the effect of different exercise amounts indistinguishable. Alternatively, it may suggest that individual athletes respond to the same high levels of exercise differently with certain athletes having an intrinsic susceptibility to ventricular arrhythmia such as concealed cardiomyopathy or electrophysiological disease which was undetectable on ECG and cardiovascular magnetic resonance (CMR).

It is unclear whether high levels of exercise itself can induce fatal ventricular arrhythmia or are merely a trigger for arrhythmogenesis in those with an underlying predisposition. Exercise-induced RV dysfunction has been shown to be associated with ventricular arrhythmia in endurance athletes with RV remodelling typically seen in athlete’s heart.^14^ Suppression of certain ventricular arrhythmias in highly trained athletes is also found to occur with de-training which directly implicates exercise-induced cardiovascular adaptations as a potential cause for these arrhythmias, although they are generally regarded as benign.^15^ In younger and middle-aged athletes, exercise training type, intensity, and experience do not appear to influence the prevalence of ventricular arrhythmia.^11,16^ Whilst our findings in older athletes are in keeping with this, without control data, we are unable to assess whether exercise itself is the substrate for ventricular arrhythmia in veteran athletes.

We did not find any significant differences in exercise habits the month prior to the onset of ventricular arrhythmia compared with the whole follow-up period. This has not previously been specifically studied in healthy athletes. However, a previous study found that heart failure patients with ICDs exhibited a decline in physical activity on accelerometers in the 2 weeks prior to a sustained ventricular arrhythmic event.^17^ Unlike those patients, we did not find a similar reduction in exercise regimes or performance prior to an arrhythmic event implying the absence of intercurrent illness in the preceding weeks to the onset of ventricular arrhythmia. Conversely, it also suggests athletes were not training more often or intensely compared with their baseline to influence the onset of ventricular arrhythmia.

### Myocardial fibrosis as a possible substrate for ventricular arrhythmia

Athletes who experienced ventricular arrhythmia had a significantly greater prevalence of myocardial fibrosis than athletes without ventricular arrhythmia. Myocardial fibrosis is known to be arrhythmogenic in several non-ischaemic cardiomyopathies, and we have previously shown it to be independently associated with the risk of developing ventricular arrhythmia amongst this cohort.^5,18^ It is therefore highly plausible that it may have served as the substrate for arrhythmogenesis within this particular cohort.

Although athletes with and without ventricular arrhythmia had no overall differences in training volume or intensity, all athletes with sustained VT had myocardial fibrosis on CMR and each of these events occurred during exercise. Therefore, it is possible that myocardial fibrosis may lower the arrhythmic threshold during acute exercise activity in certain athletes.

### Timing and characteristics of arrhythmia in relation to exercise

All three cases of sustained VT occurred during or within 1 h of exercise. This further strengthens the notion of malignant ventricular arrhythmia being associated with acute exercise activity in athletes and underlines the importance of automated external defibrillator availability in sport settings along with adequate resuscitation training. In young Italian athletes who suffered SCD, the risk of exercise-related SCD was demonstrated to be greater in athletes than non-athletic individuals, particularly in those with pre-existing underlying cardiac disease.^19^ Whilst our study was not powered to detect SCD, the majority of arrhythmic events occurred closer to exercise and therefore further suggests the presence of a possible underlying cardiac substrate within our cohort.

However, a significant proportion of ventricular arrhythmia occurred outside of the time frame considered as ‘sports-related’ typically used to categorize the timing of SCD as 21.8% occurred over 24 h since the last exercise session.^20^ Furthermore, almost 10% of ventricular arrhythmia occurred nocturnally. Previous studies have found a significant number of athletes suffer SCD during sleep.^3,21^ In a study by Finocchiaro *et al.* where the majority of 357 athletes who suffered SCD had structurally normal hearts on post-mortem, 40% of SCD cases occurred outside of physical exertion.^3^ Hence, it is debated that periods of rest, where extreme bradycardia and QT interval prolongation occur, may precipitate ventricular arrhythmia and thus SCD.^22^ However, there is a paucity of data regarding the detailed circumstances and timing of SCD and sudden cardiac arrest (SCA) in relation to exercise activity, particularly in veteran athletes.

### The clinical utility of premature ventricular contractions

Athletes who developed ventricular arrhythmia had a significantly greater prevalence of PVCs during exercise testing including those with atypical features. Furthermore, the presence of PVCs during exercise had the greatest sensitivity for identifying athletes who developed ventricular arrhythmia closer to exercise activity. These findings support the clinical practice of exercise testing in athletes at risk of ventricular arrhythmia, particularly as all three athletes who developed sustained VT exhibited PVCs with atypical features during exercise testing.^2^ This association of atypical PVCs with an increased risk of ventricular arrhythmia is in keeping with a study by Compagnucci *et al.* who found that uncommon PVC morphology was associated with a greater risk of sustained ventricular arrhythmia.^10^ However, we also found that that PVCs during exercise testing were highly prevalent irrespective of ventricular arrhythmia status and had the lowest sensitivity for ventricular arrhythmic events occurring over 24 h since exercise.

Our findings emphasize the importance of assessing for PVCs during exercise alongside other clinical factors such as myocardial fibrosis. Furthermore, asymptomatic NSVT occurred first in all athletes who subsequently developed symptomatic VT highlighting the key role of screening for NSVT including outside of exercise and in particular during sleep. The combination of atypical PVCs or NSVT with myocardial fibrosis on CMR should certainly alert physicians to the potential increased risk of ventricular arrhythmia in veteran athletes.

### Exercise tracking in athletes

The use of exercise tracking of athletes is a relatively novel concept within the scientific literature.^23^ In the field of Sports Cardiology, measurement of prospective exercise activity presents an important opportunity to assess a key variable which is central to the methodology of many studies. Most existing studies have relied upon self-reported training histories which may be unreliable leading to an inaccurate representation of study populations. In our study, we not only measured formal exercise but were also able to quantify general physical activity via accelerometers within ILRs.

We specified an inclusion criterion of in excess of 10 h of weekly exercise training. The average weekly self-reported training history of our cohort was over 11 h per week whilst we found the average prospectively tracked exercise amount was 7.2 h per week which is considerably less than the self-reported figure. Whilst it is possible that exercise levels reduced in those previously training over 10 h per week, it also suggests that certain athletes may over-report the amount of exercise they perform. The use of CETDs in our study provides key insight into the discrepancy between self-reported training histories and actual exercise activity in athletes. Our findings suggest that future prospective studies involving athletes should incorporate prospectively tracked exercise activity or as a minimum use automated training diaries linked to CETDs to screen athletes using their existing data to ensure reported exercise levels are accurate.

### Limitations

Our study was limited by the highly selected cohort which exclusively consisted of White European veteran male cyclists and triathletes which limits clinical translation to other groups and does not allow for meaningful comparison of different sporting activities. Furthermore, athletes were recruited via self-referral and it is possible that certain athletes took part in the study for medical assessment of symptoms which they did not disclose. When assessing the impact of exercise volume and intensity, athletes used their own tracking equipment which may have led to inconsistencies in the generated data. Also, multiple events were included from individual athletes which may have introduced bias. Whilst we classified NSVT according to consensus guidelines of broad complex tachycardia of ventricular origin of equal or greater than three beats, it may be argued that a longer minimum number of beats would be more clinically significant as the association of short runs of asymptomatic NSVT with SCD in athletes is unclear. Furthermore, ILR recordings consisted of single lead ECG and 12-lead ECG during exercise was not performed, thus making it not possible to determine the site of origin of PVC and ventricular arrhythmia. Furthermore, an electrophysiology study with electro-anatomical mapping was not systematically performed, at a difference with prior published experiences, which may have helped characterize the type of ventricular arrhythmia.^24^ Finally, the minimum high ventricular detection on ILR’s was eight beats which may have led to episodes shorter than this being missed in certain athletes.

## Conclusion

In male veteran endurances athletes, myocardial fibrosis was strongly associated with the risk of ventricular arrhythmia. Furthermore, the presence of atypical PVCs during exercise testing was specific to athletes who developed ventricular arrhythmia, thus emphasizing the clinical importance of screening for both of these features. Specific differences in exercise training patterns did not influence the overall incidence or timing of ventricular arrhythmia. However, sustained VT exclusively occurred during exercise in athletes with myocardial fibrosis suggesting that myocardial fibrosis may be an arrhythmogenic substrate which is activated by intense exercise in certain veteran athletes. However, larger studies involving control data are needed to confirm this.

## Supplementary Material

zwag021_Supplementary_Data

## Data Availability

Data will be made available on request to the corresponding author.

## Presentations

EAPC Annual Congress 2025-Young Investigator Award Final (Sports Cardiology)
