# Supplementary material for: The timing and relationship of ventricular arrhythmia with exercise patterns in veteran male endurance athletes
Source: Eur J Prev Cardiol. 2026 Jan 12;33(8):1427–37. doi: 10.1093/eurjpc/zwag021 (PMC13229576; doi:10.1093/eurjpc/zwag021)
Supplement: zwag021_Supplementary_Data [file zwag021_supplementary_data.docx]

**Supplemental Material**

Table of Contents

[Supplemental Results 2](#_Toc217237436)

# Supplemental Results

Table 1; Subgroup Analysis of Athletes with Ventricular Arrhythmia According to the Presence of Myocardial Fibrosis

|  | **Athlete Without Fibrosis (n=6)** | **Athlete With Fibrosis (n=19)** |
| --- | --- | --- |
| **Baseline Characteristics** | | |
| Age (Years) | 54.9 ± 2.2 | 61.6 ± 5.6 |
| Systolic Blood Pressure (mmHg) | 120.3 ± 11.4 | 116.2 ± 10.4 |
| Diastolic Blood Pressure (mmHg) | 77.0 ± 8.3 | 71.4 ± 6.6 |
| Resting Heart Rate (BPM) | 54.2 ± 7.1 | 51.2 ± 5.0 |
| **Self-Reported Training History** | | |
| Training Years (> 10 Hours/Week) | 12.8 ± 4.4 | 18.5 ± 10.8 |
| Weekly Training (Hours) | 11.8 ± 2.5 | 10.9 ± 1.9 |
| Competitions Per Year | 17.3 ± 11.2 | 21.5 ± 12.1 |
| **Exercise Test** | | |
| FTP (W) | 257.6 ± 24.4 | 248.1 ± 30.5 |
| PVCs During Exercise Testing (n) | 4 (66.7%) | 17 (89.5%) |
| Atypical PVCs During Exercise Testing (n) | 2 (33.3%) | 12 (63.2%) |
| **CMR** | | |
| LVEDVi (ml/m^2^) | 103 ± 14 | 117 ± 17 |
| LVEF (%) | 53.8 ± 4.4 | 56.3 ± 4.1 |
| LVMi (g/m^2^) | 69.8 ± 9.7 | 72.7 ± 10.2 |
| RVEDVi (ml/m^2^) | 104 ± 15 | 117 ± 17 |
| RVEF (%) | 50.9 ± 5.2 | 52.5 ± 6.8 |
| Native T1 (ms) | 1236 ± 34 | 1254 ± 51 |
| ECV (%) | 19.7 ± 1.3 | 21.5 ± 2.0 |
| T2 (ms) | 40.2 ± 1.3 | 41.1 ± 2.5 |
| **Prospective Exercise Data (Monthly)** | | |
| Exercise Frequency (n) | 14.0 (9.8 – 18.3) | 16.7 (13.4 – 20) |
| Exercise Duration (hours) | 34.6 ± 20.8 | 30.3 ± 9.5 |
| Exercise Distance (miles) | 441 (68 – 815) | 481 (367 – 596) |
| Exercise Intensity (TSS) | 1584 (844 – 2325) | 1587 (1339 – 1836) |
| **Ventricular Arrhythmic Event** | | |
| Sustained VT (n) | 0 | 3 (15.8%) |
| >1 Arrhythmic Event (n) | 2 (33.3%) | 11 (57.9%) |
| Arrhythmia Rate (BPM) | 214.2 ± 22.4 | 201.0 ± 36.4 |
| Arrhythmia During Exercise (n) | 1 (16.7%) | 8 (42.1%) |
| **Power Zones (% of time spent)** | | |
| **1** | 30.5 (14.8 – 46.2) | 35.4 (23.5 – 47.4) |
| **2** | 28.0 (14.0 – 42.1) | 28.1 (20.8 – 35.3) |
| **3** | 16.3 (13.8 – 18.9) | 13.4 (7.5 – 19.3) |
| **4** | 8.6 (3.9 – 13.4) | 9.1 (6.4 – 11.8) |
| **5** | 3.4 (0.0 – 8.6) | 4.2 (2.7 – 5.6) |
| **6** | 2.9 (0.0 – 10.6) | 3.4 (1.1 – 5.7) |
| **Heart Rate Zones (% of time spent)** | | |
| **1** | 53.7 (27.8 – 79.5) | 47.4 (33.2 – 61.5) |
| **2** | 15.4 (9.8 – 20.9) | 22.3 (14.6 – 30.0) |
| **3** | 8.4 (0.8 – 16.0) | 12.3 (7.2 – 17.4) |
| **4** | 7.7 (1.9 – 13.6) | 8.7 (5.5 – 11.8) |
| **5** | 2.8 (0.5 – 5.1) | 1.9 (0.7 – 3.2) |
| **6** | 2.8 (0.0 – 12.1) | 1.2 (0.0 – 5.5) |

Values are mean ± standard deviation or frequency (%) or median (interquartile range). * P<0.05. Abbreviations: BPM, beats per minute; CMR; cardiac magnetic resonance; ECV, extracellular volume; FTP, functional threshold power; LAVi, left atrial volume indexed; LVEDVi, left ventricular end-diastolic volume indexed; LVEF, left ventricular ejection fraction; LVMi, left ventricular mass indexed;; RVEDVi, right ventricular end-diastolic volume indexed; RVEF, right ventricular ejection fraction; TSS, training stress score; VT, ventricular tachycardia.
